# Cycle Threshold Value as a Predictor of Severity and Intensive Care Unit Admission for Children Presenting to the Emergency Department With COVID-19: A Single-Center Experience From Saudi Arabia

**DOI:** 10.7759/cureus.26614

**Published:** 2022-07-06

**Authors:** Ali S Al-Shareef, Bader Shirah, Mohammed Dabroom, Mohammed M Ahmed, Khalid A Aljohani, Mona A Al Dabbagh, Majid Ramadan, Fahad Hakami

**Affiliations:** 1 Emergency Medicine, King Abdulaziz Medical City - Western Region, Jeddah, SAU; 2 Medicine, King Saud Bin Abdulaziz University for Health Sciences, Jeddah, SAU; 3 Clinical Research, King Abdullah International Medical Research Center, Jeddah, SAU; 4 Neuroscience, King Faisal Specialist Hospital and Research Centre, Jeddah, SAU; 5 Medicine, Ibn Sina National College, Jeddah, SAU; 6 Pediatrics, King Saud Bin Abdulaziz University for Health Sciences, Jeddah, SAU; 7 Pediatric Infectious Diseases and Transplant Infectious Diseases, King Abdulaziz Medical City, National Guard Health Affairs, Jeddah, SAU; 8 Public Health, King Abdullah International Medical Research Center, Jeddah, SAU; 9 Pathology and Laboratory Medicine/Genetics, King Abdulaziz Medical City - Western Region, Jeddah, SAU

**Keywords:** saudi arabia, infection, children, coronavirus, sars cov-2, covid-19

## Abstract

Introduction: The alarming infection rates of COVID-19 and variability in disease severity and outcome created the need for a prognostic marker to predict disease severity, prioritize services, and assist in clinical decision-making. The cycle threshold (Ct) value was hypothesized to be inversely correlated with viral load and subsequently disease severity. Therefore, it gained clinical interest and was an important topic for research. In this study, we aimed to determine the accuracy of the Ct value as a predictor of clinical severity in children presenting to the emergency department with COVID-19. Specifically, we aimed to compare the relationship between clinical severity among patients with high vs. low Ct values as well as to assess the correlation between the mean Ct value with the mean number of symptoms.

Methods: This is a single-center retrospective cohort study. Data were obtained from the electronic medical record software of King Abdulaziz Medical City in Jeddah, Saudi Arabia. The present study included randomly selected COVID-19 cases aged ≥1 months to 18 years who presented to the emergency department between March 2020 and May 2021. Collected clinical data were matched with laboratory data at the time of diagnosis to examine the association between Ct values and clinical factors.

Results: A total of 191 COVID-19 PCR-positive children were included with an overall mean Ct value of 11.5, a median of 10, and a highest Ct value of 25. The mean age of the patients was 95 months. More than half (51.35%) of the patients were admitted to the hospital, while 2.09% were admitted to the intensive care unit and one patient (0.52%) died. There was no significant association between Ct values and demographics or clinical characteristics of the patients.

Conclusion: We demonstrated a lack of association between SARS-CoV-2 Ct value detected in nasopharyngeal swabs with disease severity, number of symptoms, oxygen requirement, intensive care unit admission, or length of hospital stay in the pediatric population presenting to the emergency department with COVID-19. This finding does not support the routine reporting of Ct values to aid clinicians in making clinical and patient-management decisions for COVID-19 patients or guide infection control or public health decisions. Further studies confirming our observations are needed.

## Introduction

Clinical medicine in Saudi Arabia and countries around the world has been transformed by the coronavirus disease 2019 (COVID-19) pandemic, which is caused by the SARS-CoV-2 [1]. On March 11, 2020, this disease was declared a global pandemic by the World Health Organization (WHO) [2]. Since that time, the virus spread rapidly worldwide and as of June 29, 2022, there were more than 542 million cases around the world, and more than 792,000 confirmed cases in Saudi Arabia [3]. The Saudi government implemented a curfew in earlier months of the pandemic to protect both citizens and expatriates and to limit the spread of this highly infectious virus given the need for strict social isolation [4]. Interim guidelines, infection prevention and control measures, and policies were implemented to limit disease spreading nationwide [4].

Among the healthcare systems in Saudi Arabia to be directly affected by COVID-19 was King Abdulaziz Medical City-Jeddah, a 751-bed medical complex. It was prepared to provide healthcare services for a large number of COVID-19 inpatients while continuing to provide clinical care for its regular patients. Previous studies have shown that children are less affected by COVID-19 as compared to adults. Furthermore, affected children may be asymptomatic or too mildly infected to draw medical attention and be tested and counted in observed cases of COVID-19 [5]. This rapid spread in pandemic proportions caused an abrupt rise in the demand and significant strain on healthcare services.

SARS-CoV-2 infection is diagnostically confirmed through real-time polymerase chain reaction (RT-PCR), which is considered to be the gold standard [6,7]. With high infection rates and variability in disease severity and outcome, there was a need for a prognostic marker to predict disease severity, prioritize services, and assist in clinical decision-making. Among those considered, the cycle threshold (Ct) gained clinical interest as it was hypothesized to be inversely correlated with viral load and subsequently disease severity. This interest has peaked as multiple studies reported that a lower Ct value indicating higher viral load can be associated with disease severity and outcome [8-10]. Other studies did not show association [11]. Studies examining SARS-CoV-2 RNA levels in children, which generally involved small sample sizes, have mostly agreed that symptomatic subjects had lower Ct values [12,13].

The literature is surfacing with mixed reviews and analyses on the association between Ct value and clinical severity in COVID-19 patients. Studies reporting a positive association between the concerned variables of this study (i.e. Ct value and clinical severity of COVID-19) are many, but the most striking study was by Rao reporting data that demonstrates a positive association between Ct value and severity of the disease [6]. Another very recent study adds to the literature that there is a positive association between low Ct values and the severity of COVID-19. The data comprised more than 34,000 COVID-19-positive patients [14]. A detailed study conducted on children as well as adults also reported an inversely proportional relationship between Ct values and the severity of some respiratory viruses [15]. A study done on samples of nasopharyngeal swabs from the population of children having positive COVID-19 concluded an inversely proportional relationship between Ct values and lower viral loads. Moreover, it added that children are not playing the main role in the transmission process of the virus [16]. Liu et al. also found an association between viral load (low Ct value) and disease severity [17]. Another study supporting the evidence that Ct values are associated with the severity of the diseases was done by Mowrer [18] revealing that patients with high Ct values were lacking the transmission events.

The data on the pediatric population revealing no association between Ct value and the severity of the disease is scarce. The literature body is evidence of having studies that show zero association between Ct value and severity of the disease of COVID-19, but these studies have not included the pediatric population. A cross-sectional study done by Strutner and colleagues compared Ct values in symptomatic vs. asymptomatic children and found that symptomatic children, especially those <5 years of age, had significantly lower Ct values compared to asymptomatic children, suggesting that higher viral load plays a role in symptom development [19]. Similar findings were demonstrated in a community-based study of 555 children and adults where symptomatic subjects had significantly lower Ct values (with higher SARS-CoV-2 RNA levels) compared with asymptomatic individuals [20]. On the other hand, a study done in Saudi Arabia on adults and children failed to demonstrate any association between the Ct value and the criticality of illness [21].

A study done in India included 219 patients who tested positive for COVID-19. The sample size was divided into four groups (patients with severe disease vs. patients with mild disease, patients who survived vs. patients who died). The author collected data on Ct values and compared them within both groups. The analysis found low Ct values in the patients with mild disease as compared to the opponent group (i.e., patients having a severe disease). Moreover, patients who were on the “died” list showed relatively lower Ct values as compared to those patients who survived, hence this study reported no correlation between Ct value and severity of the disease [22].

In this study, we aimed to measure the accuracy of the Ct value as a predictor of clinical severity in the pediatric population presenting to the emergency department with COVID-19. Specifically, we aimed to compare the relationship between clinical severity among patients with a high vs. low Ct value as well as to assess the correlation between the mean Ct value with the mean number of the symptoms.

## Materials and methods

Data sources

The current study is a single-center retrospective cohort study using data from the electronic health record system of King Abdulaziz Medical City in Jeddah, Saudi Arabia. The study included all pediatric COVID-19 cases who were presented to the emergency department between March 2020 and May 2021. Demographics and clinical data were collected from the selected cases. To examine the association between Ct values and clinical factors, collected clinical data were matched with laboratory data at the time of diagnosis. Clinical data of each case were connected to its respective Ct values upon admission. All cases were diagnosed as COVID-19 based on the Abbott RT-PCR tests of nasopharyngeal samples. The Abbott PanBio was recommended by World Health Organization due to its high sensitivity (95% CI: 79.0%-92.0%) and specificity (95% CI: 99.4%-100%). A study indicated that the sensitivity was 95.8% in Ct-values <25 and within the first seven days from symptom onset [23]. Moreover, another study confirmed that nasopharyngeal specimens are more reliable than buccal specimens, especially for screening of COVID-19 in children [24].

Identification of patients

All individuals aged ≥1 month to 18 years who tested positive for SARS-CoV-2 virus and visited the emergency department between March 2020 and May 2021 were included in the analysis. The case definition of COVID-19 suspected cases included three categories. The first category included patients with acute respiratory illness (sudden onset of at least one of the following: fever (measured or by history), cough, or shortness of breath). The second category included patients with sudden onset of at least one of the following: headache, sore throat, rhinorrhea, nausea, or diarrhea, and in the 14 days prior to symptom onset, either having contact with a confirmed COVID-19 case or living in a facility known to be experiencing an outbreak of COVID-19. The third category included an unexplained severe acute respiratory infection, either community-acquired pneumonia or hospital-acquired pneumonia in a recently discharged patient. An additional group included children who were asymptomatic but identified as part of contact tracing. The definition of the COVID-19 confirmed case was a person who met the suspected case definition with laboratory confirmation of SARS CoV-2 infection by PCR.

Admission criteria

Early in the pandemic, we used to admit all patients even if asymptomatic and then the hospital protocol changed to admit patients only if they met the case definition of confirmed/suspected COVID-19 who are symptomatic plus any of the high-risk criteria. High-risk criteria for hospitalization included infants aged <1 year, clinical or radiographic evidence of pneumonia, SpO2 <92% on room air, respiratory failure, preexisting chronic conditions such as uncontrolled asthma, immunosuppressed patient, clinical features that are similar to those of toxic shock syndrome, atypical Kawasaki disease, gastroenteritis with dehydration and/or poor oral intake, persistent high fever lasting for three to five days, disease course longer than one week with no improvements in symptoms or signs of progressive exacerbation, severe obesity (body mass index ≥120% of the 95th percentile), or not applicable home isolation or non-hospital-based quarantine facilities.

Study variables

Patient demographics and clinical characteristics including age, gender, current medications, frequency of symptoms, treatment, chest x-ray findings, patient disposition, length of stay in the hospital, and reported complication were included in the analysis as potential predictors of low Ct values. The main outcome of interest was clinical severity, represented by the need for oxygen support, intensive care unit admission, and mortality. To obtain balanced and statistically optimal comparable groups, we used a Ct value of 10 as a cut-off point for our analysis. The study was approved by the Institutional Review Board (IRB) in King Abdullah International Medical Research Center (KAIMRC) (IRB number is IRBC/0830/21).

Statistical analysis

The demographic and clinical characteristics were assessed across determined Ct values using unadjusted chi-square and Fisher exact test for categorical variables and t-test for numeric variables. We used an adjusted multivariate generalize the linear model to estimate predictors for Ct values as a continuous outcome. The assumptions of normality and homogeneity of variance were examined using the Akaike information criterion (AIC). No high AIC was observed. All statistical tests were two-sided, and findings were considered statistically significant at P<0.05. All analyses were conducted using SAS statistical software version 9.4 (SAS Institute Inc. Cary, NC).

## Results

**Figure 1:**
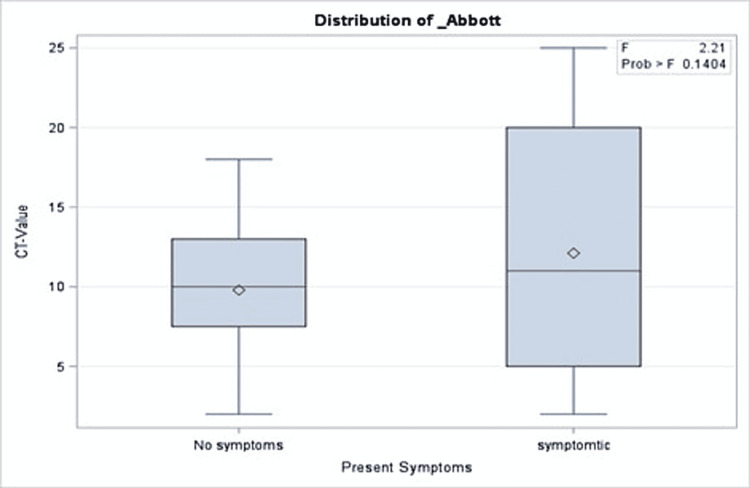
Results of multivariate analysis assessing the Ct values as a predictor for the number of presenting symptoms in children presenting to the emergency department with COVID-19

**Figure 2:**
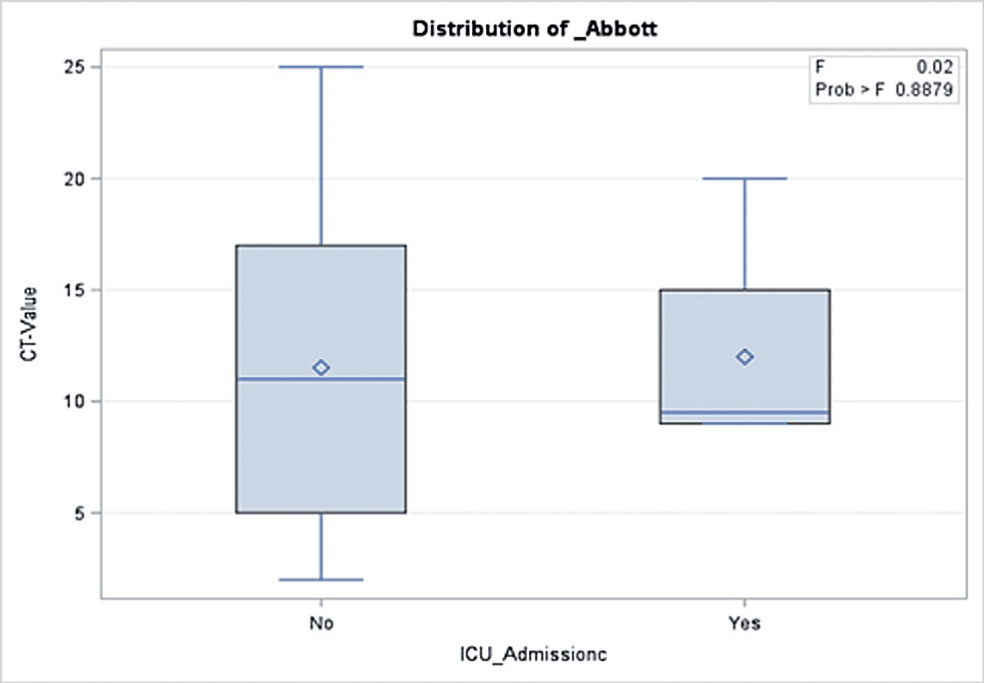
Results of multivariate analysis assessing the Ct values as a predictor for ICU admission in children presenting to the emergency department with COVID-19

**Figure 3:**
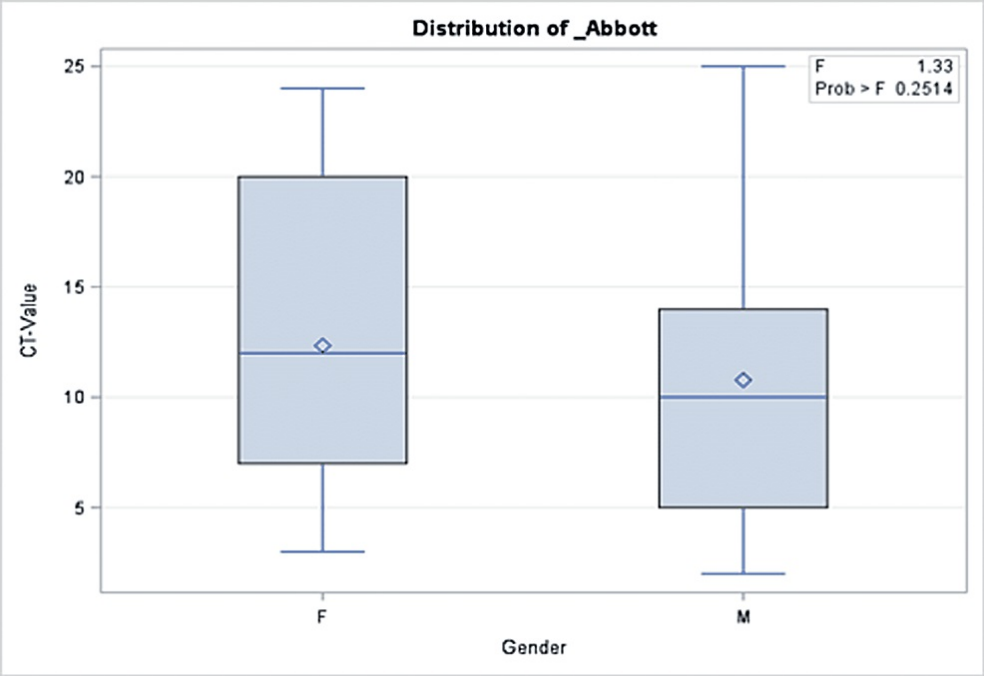
Results of multivariate analysis assessing the relationship between Ct values and gender in children presenting to the emergency department with COVID-19

**Figure 4:**
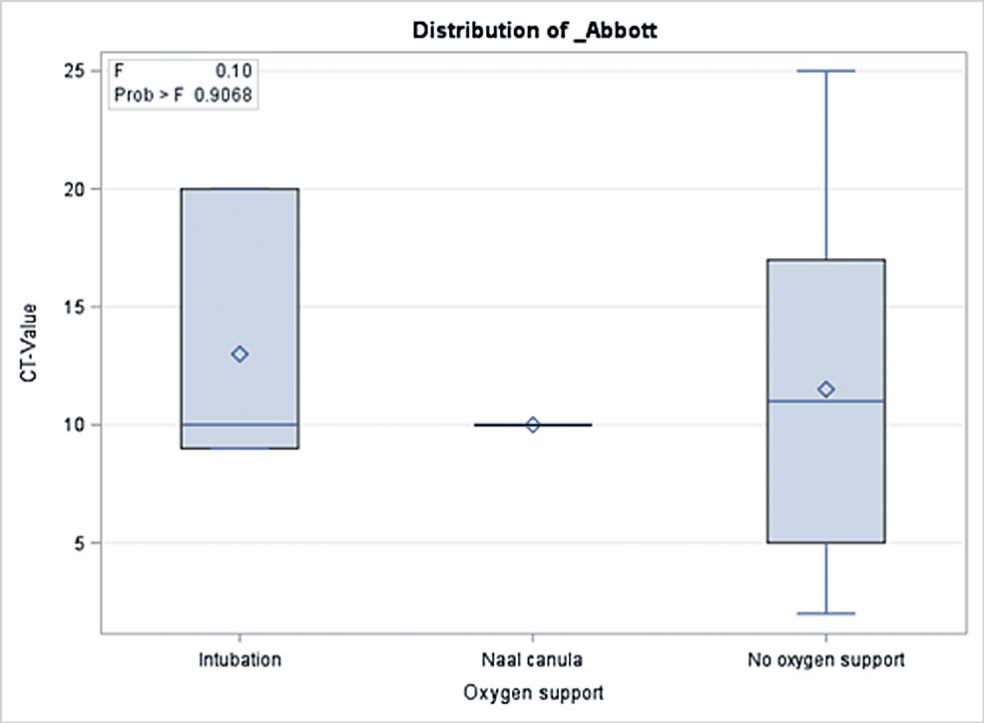
Results of multivariate analysis assessing the Ct values as a predictor for oxygen support in children presenting to the emergency department with COVID-19

**Figure 5:**
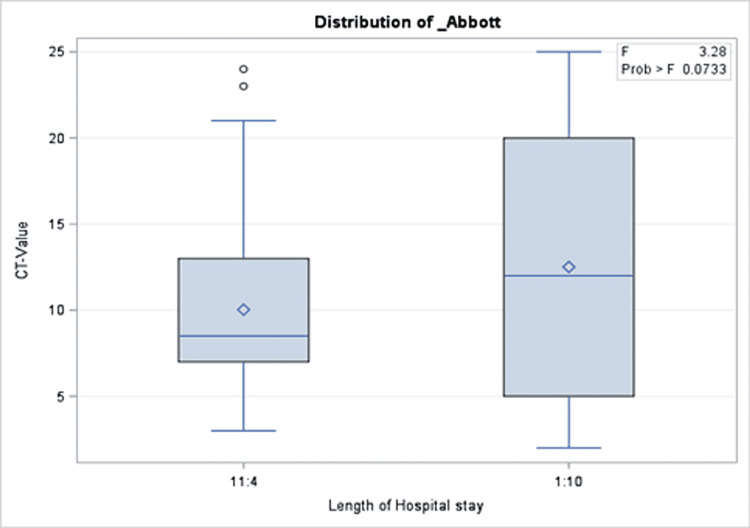
Results of multivariate analysis assessing the Ct values as a predictor for length of hospital stay in children presenting to the emergency department with COVID-19

A total of 191 COVID-19 PCR-positive children were included with an overall mean Ct value of 11.5, a median of 10, and 25 as the highest Ct value recorded. The mean age of the patients was 95 months. More than half of the patients (51.35%) were admitted to the hospital, while 2.09% were admitted to the intensive care unit and one patient (0.52%) died. Approximately one out of three patients were taking medication before the COVID-19 infection period. Similarly, 30.89% and 31.93% of the cohort received COVID-19 treatment and had comorbidities during the infection, respectively. More than half of the cohort (65.96%) reported a median of two COVID-19 symptoms during their infection. The percentages of those who had complications and those who needed oxygen support were 2.09% and 2.08%, respectively (Table 2). There was no significant association between Ct values and demographics or clinical characteristics of the patients (Tables 1, 2). Precisely, both groups with a Ct value of lower or higher than 10 had close proportions of reported symptoms (64.29% and 67.74%, respectively; p = 0.61). Similarly, those with Ct values=<10 compared to those with higher values were not different with regard to their need for oxygen support in (6.12% vs. 1.96%; p = 0.48), intensive care unit admission rate (2.09% vs. 3.06%; p = 0.33), and mortality (0% vs. 0.52%, p = 0.303). The same patterns were observed for all other clinical and hospital-related factors as shown in Table 2. In the multivariable model, there was no significant association between Ct values and presenting symptoms, intensive care unit admission, oxygen requirements, or length of hospital stay (Figures 1-5).

**Table 1:** Demographic details of children presenting to the emergency department with COVID-19 CNS: Central nervous system. * Earlier in the COVID-19 pandemic all identified patients with positive SARS-CoV2 PCR as part of contact screening were admitted to the hospital. Thus, these numbers include many admitted patients that were asymptomatic. ** These exclude asthma and hyperactive airway disease (which are included in the chronic respiratory condition). *** one case had autism spectrum disorder and one had warts.

Characteristics	# of patients	Ct value (<=10) No. (%)	CT value >10 No. (%)	P value
Total	191	98 (51.31)	93 (48.69)	
Age (months)				0.54
Mean	95.17	84	78	
Median	84	2	2	
Range	84-227	84-228	78-228	
Age				0.14
< 12 months		26 (26.53)	28 (30.11)	
1 to 5 years old		46 (46.94)	34 (36.56)	
6 to 10 years old		24 (24.49)	23 (24.73)	
11 to 18 years old		2 (2.04)	8 (8.6)	
Gender				0.21
Male	95 (49.73)	53 (54.08)	42 (45.16)	
Female	96 (50.26)	45 (45.92)	51 (54.84)	
Underlying medical condition				
No comorbidity	130			
Chronic respiratory condition	16			
Diabetes mellitus	1			
Other endocrine disorders	3			
Prematurity	1			
Cardiovascular disorder	15			
Active malignancy, hematological malignancy, or solid tumors	14, 12, 2			
Hematological disorder	4			
Neurological disorder	4			
Gastrointestinal disorder	2			
Metabolic/storage disorder	2			
Obesity	1			
Allergic disorder**	5			
Kawasaki disease	1			
Others***	2			
Patient Disposition*				0.503
Admission	100 (52.35)	49 (50.00)	51 (54.84)	
Home isolation	91 (47.64)	49 (50.00)	42 (45.16)	
Current medications				0.48
Yes	58 (30.36)	32 (32.65)	26 (27.96)	
No	133 (69.63)	66 (67.35)	67 (72.04)	

**Table 2:** Clinical characteristics and outcome of children presenting to the emergency department with COVID-19

Characteristics	# of patients	Ct value =<10 No. (%)	Ct value >10 No. (%)	P value
Total	(n=191)	98	93	
Presenting symptoms				0.61
Yes	126 (65.96)	63 (64.29)	63 (67.74)	
No	65 (34.03)	35 (35.71)	30 (32.26)	
Number of symptoms				0.35
Mean	1.73	1.71	1.75	
Median	2	2	2	
Range	1-7	1-6	1-7	
Comorbidity				0.25
Yes	61 (31.93)	35 (35.71)	26 (27.96)	
No	130 (68.06)	63 (64.29)	67 (72.04)	
Chest X-ray findings				
Diagnostic	18 (9.42)	10 (10.2)	8 (8.6)	0.62
Normal	34 (17.8)	14 (14.28)	20 (21.5)	
No X-ray	139 (72.77)	74 (75.51)	65 (69.89)	
Oxygen support				0.48
Intubation	2 (1.04)	2 (4.08)	0	
Nasal canula	2 (1.04)	1 (2.04)	1 (1.96)	
No oxygen support	187 (97.91)	95 (93.88)	92 (98.04)	
Intensive care unit admission				
Yes	4 (2.09)	3 (3.06)	1 (1.08)	0.33
No	187 (97.91)	95 (96.94)	92 (98.92)	
Length of hospital stay (days)				0.09
1-10 days	59 (30.89)	24 (24.49)	35 (37.63)	
11-40 days	40 (20.94)	25 (25.51)	15 (16.13)	
No admission	92 (48.16)	49 (50.00)	43 (46.24)	
Treatment				0.13
Yes	59 (30.89)	35 (35.71)	24 (25.81)	
No	132 (69.11)	63 (64.29)	69 (74.19)	
Outcome				0.303
Death	1 (0.52)	0	1 (1.08)	
Recovered	190 (99.47)	98 (100)	92 (98.92)	

## Discussion

Our single-center retrospective cohort study from a large tertiary care center in Saudi Arabia found no significant association between lower Ct values and disease severity evident by the clinical characteristics of the disease, number of symptoms, oxygen requirement, intensive care unit admission, and length of hospital stay. This study was unique in that participants represented the entire spectrum of COVID-19 in the pediatric population ranging from asymptomatic infection to lethal disease. Our results align with that of Shah's study [22] because in their study they also were unable to find the association between Ct values and disease severity and/or deaths in patients with COVID-19. A very striking commentary on the Ct value as a predictor of viral load adds to the literature that the Ct value obtained from qualitative PCR test cannot be used as a quantification unit. The article adds that the Ct value itself is not enough to depict the viral load unless and until it has been used with a standard curve along with the references [25]. The data analysis in our research has provided evidence that there has been no relationship observed between Ct value and severity in disease, particularly in the pediatric population.

Our findings oppose what is reported in the literature. A systemic review conducted early during the pandemic reported that the majority of studies showed an association between low SARS-Cov2 Ct values and worse clinical outcomes, suggesting that Ct value is useful in predicting the clinical outcome and prognosis of COVID-19 patients. This review, however, included adult subjects only [6]. In addition, one study found an inverse correlation between Ct values and severe cases, especially at the time of admission, and in this study, severe patients’ characteristics determined shifting to the intensive care unit along with other conditions [17]. Another study done on 192 patients who tested positive for COVID-19 revealed a positive and inverse correlation between Ct values and hospital admissions, intensive care unit admission, intensive care unit stay length, and death rates [26]. As far as the relation between Ct values and symptoms is concerned, a study found a significant correlation between Ct values and the symptoms. The author found that in patients with no symptoms, the Ct values were higher compared to those who were having symptoms, thus it can be said that Ct values have an inversely proportional relationship with symptoms of COVID-19 [27]. Although a recently published meta-analysis of seven studies on adults found no significant difference in mean Ct values between hospitalized and non-hospitalized patients. The study found that among hospitalized patients, those with lower Ct values had a higher risk for more severe disease and mortality compared to those with higher Ct values.

Few experts in the field argue that Ct values are subjected to variability due to the usage of different kits and even techniques (e.g., fluorescence threshold values or targeted genes). Moreover, it also varies between the analysis (runs) of the same kit [25]. An important factor that should be controlled in such studies is the timing of the sample collection. The samples collected before the onset of symptoms may have lower Ct values, as compared to those samples that have been collected after the onset of the symptoms [28]. This might be one of the factors that affected our data results. This should be considered in designing future studies as it significantly impacts the results.

Another important aspect considered by researchers is the correlation of Ct value with the ability of the virus to be transmitted to another person. A very detailed and striking study done by Bullard considered data from adults as well as children and revealed that viral growth observed in the samples from children (taken through nasopharyngeal swab) was significantly lower than those samples taken from adults, depicting that children are less likely to spread the virus as compared to adults [16]. This research also supports a sense that using Ct value for isolation decisions is also falling and questionable [22].

The present study has certain limitations. Firstly, the study utilized the data from only one health care center in Saudi Arabia, therefore the need for more data is required to confirm the achieved results. In addition, the Ct value obtained for each pediatric case was extracted from the data recorded as a basic protocol for each patient upon presentation to the emergency department regardless of disease severity or duration since the onset of symptoms. Furthermore, at the beginning of the epidemic, good numbers of admitted cases were asymptomatic or had minor symptoms as part of tracing activity after contact with their infected family members which might give inaccurate association. The quality and timing of each sample may be subjected to variability in results. Moreover, the sample specifically collected for Ct value may differ in days of illness/symptom onset in each case, which again might be a reason for different results.

We urge studies to utilize much more data on a multicenter scale. The utility of the Ct value on clinical severity can be investigated in relation to additional diagnostic assays like inflammatory markers, procalcitonin, and/or lactic acid levels. Moreover, data from diverse hospital locations would also help in proving the actual fact on Ct values. The data should be time-controlled so that variations could be minimized to achieve better results. Care should be taken with sample collection timing, keeping in mind the time from the onset of symptoms. Most importantly, pediatric data have to be taken under extensive research so that the relationship between Ct value and disease severity can be revealed.

## Conclusions

Among the pediatric population presenting to the emergency department with COVID-19, we demonstrated a lack of association between SARS-CoV-2 Ct value detected in nasopharyngeal swabs and disease severity, number of symptoms, oxygen requirement, intensive care unit admission, and length of hospital stay. The current finding does not support the routine reporting of Ct values to aid clinicians in making clinical and patient management decisions for COVID-19 patients or guide infection control or public health decisions. Further studies confirming our observations are needed.
